# Canine mammary cancer diagnosis from quantitative properties of nonlinear optical images

**DOI:** 10.1364/BOE.400871

**Published:** 2020-10-16

**Authors:** Luana A. Reis, Ana P. V. Garcia, Egleidson F. A. Gomes, Francis G. J. Longford, Jeremy G. Frey, Geovanni D. Cassali, Ana M. de Paula

**Affiliations:** 1 Departamento de Física, Instituto de Ciências Exatas, Universidade Federal de Minas Gerais, 31270-901 Belo Horizonte-MG, Brazil; 2 Laboratório de Patologia Comparada, Instituto de Ciências Biológicas, Universidade Federal de Minas Gerais, 31270-901 Belo Horizonte-MG, Brazil; 3 University of Southampton, Southampton SO17 1BJ, United Kingdom

## Abstract

We present nonlinear microscopy imaging results and analysis from canine mammary cancer biopsies. Second harmonic generation imaging allows information of the collagen structure in the extracellular matrix that together with the fluorescence of the cell regions of the biopsies form a base for comprehensive image analysis. We demonstrate an automated image analysis method to classify the histological type of canine mammary cancer using a range of parameters extracted from the images. The software developed for image processing and analysis allows for the extraction of the collagen fibre network and the cell regions of the images. Thus, the tissue properties are obtained after the segmentation of the image and the metrics are measured specifically for the collagen and the cell regions. A linear discriminant analysis including all the extracted metrics allowed to clearly separate between the healthy and cancerous tissue with a 91%-accuracy. Also, a 61%-accuracy was achieved for a comparison of healthy and three histological cancer subtypes studied.

## 1. Introduction

Breast cancer is the most common type of cancer among women worldwide after only the non-melanoma skin cancer, corresponding to about 25% of new cases each year. In Brazil, this percentage is even as high as 29.7%, data from the Institute of Cancer (INCA) website [1]. However, cancer diagnosis by histological biopsy image analysis is still a challenging and demanding task for pathologists. The histological diagnosis is based on visual evaluation of the cell differentiation and the changes in the adjacent extracellular tissue [2]. Thus, methods to quantify these changes would be important diagnostic tools. Recently, it has been shown that the interplay between cells and the collagen structure and organization in the extracellular matrix (ECM) plays an important role in cancer progression and metastasis [3]. Nonlinear optical multiphoton imaging, mainly by second harmonic generation (SHG), has been increasingly used to access the collagen changes in many types of cancers. These studies have shown that the collagen properties, such as fibre alignment, density, length and thickness can be correlated with tumor progression [4–27].

In breast cancer, previous studies have shown that the increase of the collagen fibre density is correlated to some types of tumors [4,5]. The realignment of collagen at the tumor borders has also been shown to facilitate the migration of epithelial tumor cells in the process of invasion [9,19,21]. The collagen fibre organization, obtained from the anisotropy of the fibres in SHG images, has been correlated with differences between healthy and cancerous tissues and with the cancer aggressiveness [24–26]. In addition, Tao *et al.* [18] demonstrated the potential of SHG imaging for the diagnosis of fresh, unfixed surgical specimens.

Canine mammary tumors are also the most common type of cancer in female dogs, accounting for approximately 50% of the canine neoplasms [28]. It is a comparative model to human breast cancer showing epidemiological, clinical, biological and genetic similarities [28–30]. And the collagen properties obtained from SHG images have been shown to be important for prognosis [31]. However, studies quantifying both the cell and collagen properties are still lacking.

Here we present results for canine mammary cancer biopsies using multiphoton imaging by simultaneous SHG and two-photon excited photoluminescence (PL) processes. The studies where performed on standard histological biopsies stained with Hematoxylin and Eosin (H&E) to allow also for the analysis and diagnosis by the pathologists. The SHG image allows information of the collagen structure [32] in the ECM and the PL image allows to access the cell regions of the biopsy. We demonstrate an automated image analysis method to classify the histopathological types of canine mammary cancer using a range of parameters extracted from the images. A software for image processing and analysis was written in Python, including an algorithm to extract the collagen fibre network and the cell regions in the image. All the properties are obtained after the segmentation of the image into collagen and cell regions. Thus, the metrics can be measured specifically for the collagen or cell contents. A large number of parameters are obtained, including content (such as number of fibres, number of cell segments), texture (such as fibre anisotropy, SHG and PL intensity entropy) and geometric shape (such as fibre and cell network linearity and eccentricity).

These results allowed to quantify the properties of both collagen fibres and cell regions in the cancer biopsies presenting a comprehensive image analysis to identify the features that are the important ones visualized by the pathologists to classify the cancer histological types. A linear discriminant analysis (LDA) allows to clearly separate between healthy and cancerous tissues and shows a very good discrimination between the studied cancer subtypes.

## 2. Materials and methods

### 2.1 Female dog mammary tissue biopsies

We studied biopsies of mammary tissues from female dogs. The canine mammary tissue provides a good comparison to the human breast tissue and it shows the advantage of not presenting great differentiation of collagen content in the tissue throughout the life of the animal [28,33–35]. The mammary tissue samples were obtained through biopsy procedures from animals under cancer treatment at the Federal University of Minas Gerais (UFMG) Veterinary Hospital. All methods were performed in accordance to the relevant guidelines and regulations approved by the ethics committee on the care and use of animals, UFMG.

The samples in formaldehyde solution were sent to the Laboratory of Comparative Pathology, Department of General Pathology, Institute of Biological Sciences to be processed and the pathologists selected the parts for paraffin inclusion. After paraffinization, the samples were sectioned in blocks of about 4 mm sides that were cut in slices with thickness of approximately 4 to 6 μm. The tissue cuts were stained with Hematoxylin and Eosin (H&E) for the analysis and diagnosis by the pathologists. They were mounted on standard microscope slabs and covered by a thin glass coverslip.

We studied three histological subtypes, carcinoma *in situ*, carcinoma in mixed tumor and solid carcinoma, and also healthy mammary tissue for comparison. A total of 55 cases were studied, being 12 cases of carcinoma *in situ* on 17 histological slides (78 images of characteristic regions were acquired), 11 cases of carcinoma in mixed tumor on 17 slides (128 images), 19 cases of solid carcinoma on 24 slides (110 images) and 13 cases of healthy mammary gland tissue on 20 slides (100 images). The areas selected for imaging were mainly within the tumor mass.

### 2.2 Multiphoton imaging

We obtained non-linear images of the biopsies by SHG and PL in the Biophotonics Laboratory of the Physics Department at UFMG. The imaging system is a home built setup based on a confocal scanning laser unit (Olympus FV300) attached to an upright microscope (BX61 WI) using a 140 fs Ti-Sapphire oscillator (Coherent Chameleon) with 80 MHz repetition rate tuned to the wavelength of 800 nm. The sample is excited by a circularly polarized laser beam that passes through the scanning mirrors and is focused onto the sample at normal incidence to the glass coverslip by a 20× objective lens (N.A. 0.90). The average power at the sample is 7 mW, laser fluence of about $7\times 10^{-4}$ ${\mathrm{J/cm}}^{2}$. For the SHG imaging, the backscattered signal is collected by the same objective and directed by a long pass dichroic mirror (Semrock FF665-Di02), positioned just above the objective, to a non-descanned detector (a photomultiplier tube, PMT). A thin band pass filter with 20 nm bandwidth centered at the SH wavelength 400 nm (Chroma HQ400/20m-2p), together with a blocking edge filter (Semrock FF01-680/SP-25) were used in front of the detector to completely remove the laser scattered light. For the PL imaging the dichroic mirror is moved out of the beam and the backscattered signal follows the descanned path of the confocal microscope and it is measured by the internal confocal microscope PMT. The signal is filtered by a band pass filter in the range 560-600 nm and the blocking edge filter (Semrock FF01-680/SP-25). We acquired the SHG and PL images at the same sample position with areas of 0.47 mm × 0.47 mm (512 × 512 pixels). The laser transmission through the sample is also acquired as an image in a PMT positioned after the microscope condenser. The images were collected for three different focal planes, separated by 1 μm. The acquisition of the three images allowed to assure the best focal position and they were also compared by the software to eliminate noise.

### 2.3 Image analysis

We perform a comprehensive image analysis, based around segmentation of the image into regions with fibrous collagen content and those with cellular content. A software package named PyFibre (Python Fibrous Image Analysis Toolkit) was written in the Python programming language in order to perform both image segmentation and analysis, and is available on GitHub [36]. The software was developed to automate the analysis of large image data sets quickly and efficiently. Databases containing metrics extracted from the images are produced as output and can be used as quantitative properties for further analysis.

A special image format was developed for the analysis procedure, containing both SHG and PL signals, as well as a copy of the transmission data for the PL measurement. PyFibre loads in both SHG and PL images, and performs some minor pre-processing steps in order to reduce both white and shot noise. A series of further processing steps is then applied to identify fibrous and cellular regions, that utilizes the ability of the SHG signal to isolate areas of collagenous tissue. These include mapping out the locations of collagen fibres as a network using a modified version of the FIbeR Extraction (FIRE) algorithm [37] (section 2.3.1). Additional information from the PL and transmission signals is used to further refine the boundary between fibrous and cellular areas.

Sets of metrics are then calculated for each segment and averaged over to generate "global" metric databases relating to the entire image. A similar process is also carried out for metrics derived from the network that is extracted during the segmentation process. Thus, the analysis are obtained for the separated fibre and cell measured properties. These analyses can help to better understand the relationship between the cell regions and the ECM in separating the cancer histological types studied.

The calculations to quantify the organization pattern in the images are based on the structure tensor analysis [38]. A methodology already used in the literature for SHG image analysis [25,39–43]. In brief, considering the image 2D intensity map as: f(x,y), the partial derivatives along x and y contains information about the orientation of all pixels in the image [25,39–43]. The maximum and minimum eigenvalues of the structure tensor (T(x,y)), and their corresponding eigenvectors can be used to measure the image anisotropy and the fibre orientation angles. After the extracted fibre and cell networks, as presented in sections 2.3.1 and 2.3.2, the metrics can be evaluated specifically for the fibre or cell regions.

The properties analyzed (29 in total) are grouped into network, content, texture and shape. Details of the calculations are included in the software documentation [36]. In short, the metrics are: No. Fibres (Number of extracted fibres) and No. Cells (Number of cell segments); the fibre properties such as Fibre Waviness (Average fibre waviness (length / displacement)), Fibre Lengths (Average fibre pixel length), Fibre Angles (Average fibre angle), Fibre Network Degree (Average fibre network number of edges per node), Fibre Network Eigenvalue (Max eigenvalue of network adjacency matrix), Fibre Network Connectivity (Average fibre network connectivity) and Fibre Network Cross-Link Density (Average cross-links per fibre); and the fibre and cell segment properties such as Coverage (Ratio of image area containing fibres or cells to the total area), Area (Area of the image (in pixels) containing fibres or cells), Segment Shape (Average ratio of the circumference of a circle with the same area as the segment and the segment perimeter), Eccentricity (Average eccentricity of the segments), Angle SDI (Angle spectrum SDI (mean / max) for all SHG or PL segment pixels), Anisotropy (Anisotropy of structure tensor for all SHG or PL segment pixels), Local Anisotropy (Mean anisotropy of structure tensor for each SHG or PL segment pixels), SHG or PL Intensity Mean (Mean pixel intensity of SHG or PL in the fibre or cell segment), SHG or PL Intensity Standard Deviation (STD of pixel intensity of SHG or PL in the fibre or cell segment), SHG or PL Intensity Entropy (Average Shannon entropy of pixel intensities of SHG or PL in the fibre or cell segment).

#### 2.3.1 Fiber network extraction

Details of the algorithm to extract the fibre network [44] is described in the software documentation [36]. In brief, we used a modified version of the FIbeR Extraction (FIRE) software [37], that is designed to extract fibrous detail at a higher resolution than previous versions. The algorithm works by generating a network of nodes connected by edges that represent the outlines of the fibres. A primary set of nucleation points is chosen, which become parent nodes that can then each propagate subsequent child nodes in the nearby region. The main differences between our implementation and the original FIRE algorithm are the use of an edge length threshold and the ability to create an edge between any existing node, whereas originally only child nodes with the same parent node could be connected.

From the obtained network we can manipulate it either to have an overview of its structure and fibre connectivity or to obtain information about individual fibres. For that we need to identify the fibres within each network. The fibre identification is not trivial and depends on the rules assigned to the fibre definition. We apply an approach very similar to the FIRE algorithm. We identify all nodes that contain only one edge, these will definitely reside at the beginning of a fibre. These nodes become the fibre parent nodes and their first child nodes will be the only node where they share an edge.

#### 2.3.2 Fiber and cell segmentation

The extraction of fibre networks from each image allows us to obtain a rough estimate of the boundary between fibrous and cellular regions. However, in order to refine this estimation we carry out a further segmentation process based on the BDcreationHE algorithm of the CurveAlign software [45,46]. The routine involves clustering pixel vectors in RGB images and is commonly used for microscope-stained HE images, since typical gram-straining yields cellular regions of a standardised pigment. Since each 512×512 image contains $512^{2}\times 3$ data points, we employ a K-means clustering algorithm that can be performed in batch.

We attempt to mimic a HE image by generating a composite 3 channel "RGB" image from the SHG, PL and transmission signals. Each RGB pixel vector is then normalized into a unit vector, in order to reduce the dependence on the intensity of each image. Thus, collagen characteristics that have a significant SHG signal appear as unit vectors with large red components, while cellular characteristics result in unit vectors containing green and blue components. Each centroid identified by the K-means algorithm is classified as either fibrous or cellular depending on whether its mean vector lies either side of a boundary in colour space. The position of this interface is arbitrary and parameterised by sampling the data set, therefore we do not expect the algorithm to be effective in segmenting other data sets without recalculating these parameters.

Nevertheless, these combined images allowed a good separation of the regions containing fibres and cells. The procedure also provides a fully automated study of the images collected by the SHG and PL techniques that is relatively robust.

### 2.4 Linear discriminant analysis

In order to evaluate if all the metrics derived from the images enable them to distinguish between the diagnoses, we applied linear discriminant analysis (LDA). The technique is used to project a large set of data in a new space where there is a good data separability by maintaining the class discrimination [47,48].

Since LDA is a supervised clustering method, it takes into account the labels of diagnostic to look for regions where the samples will present the maximum separation. The D-dimensional input space is defined by all the 29 metrics extracted and the decision surfaces are linear functions of the input vector. Prior to performing LDA on our dataset, each feature was scaled between -1 and 1 and transformed to follow a Gaussian distribution. A stratified K-fold cross validation (CV) was applied to split our whole cohort 6 times into test and training datasets, keeping a proportional representation of each label.

### 2.5 Data statistics

One-way analysis of variance (ANOVA) was used to compare means between the diagnostic groups. For all analysis, a value p<0.05 was considered statistically significant. In this work, p values less than 0.001 are denoted by (***), p less than 0.01 are denoted by (**) and p less than (0.05) are denoted by (*).

## 3. Results and discussions

**Fig. 1.**
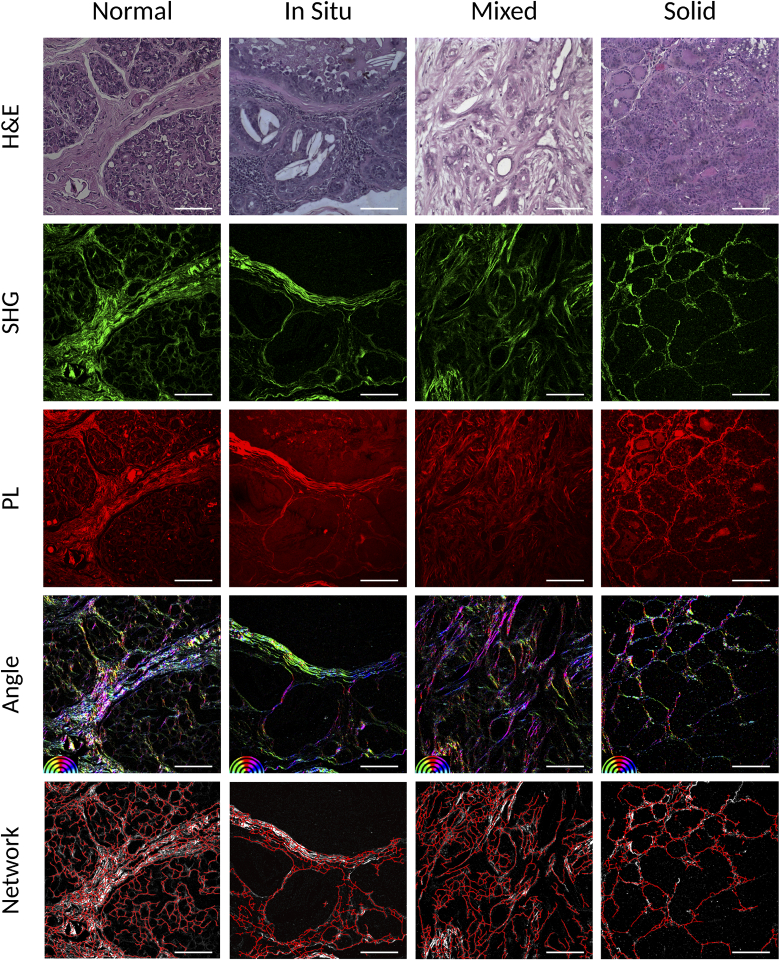
Acquired images: the H&E (first row), SHG (second row) and PL (third row) and the images extracted by the software: a color map image with the fibre angle distribution in the tissue (fourth row, angle color map at the bottom left hand side) and the collagen fibre networks in red overlapped with the SHG intensity image in gray scale (last row) for the normal tissue and each cancer type indicated in the columns as Normal, In Situ, Mixed and Solid, for the normal mammary gland, the carcinoma *in situ*, the carcinoma in mixed tumor and the solid carcinoma, respectively. The images are 0.471 mm × 0.471 mm with 512×512 pixels and the scale bar is 100 μm.

The Fig. 1 shows examples of the acquired images, in the first row are the bright field microscope images of the H&E stained tissue at roughly the same position as the acquired nonlinear images: the SHG images (at the second row) showing the collagen fibres and the PL images (at the third row) showing the eosin fluorescence. The corresponding images extracted by the software calculations [36] are presented as: the color map images of the fibre angle distribution in the tissue (fourth row) and the images of the collagen fibre networks overlapped with the SHG intensity image in gray scale (last row). The columns show the histological types labeled for short as normal, In Situ, Mixed and Solid, for the normal mammary gland, the carcinoma *in situ*, the carcinoma in mixed tumor and the solid carcinoma, respectively.

The normal mammary gland presents acini and ducts consisting of luminous epithelial cells lined by myoepithelial cells that are separated from the surrounding connective tissue by a basement membrane [49,50]. The collagen fibres, abundant in the surrounding connective tissue, are arranged in different directions throughout the mammary tissue.

The carcinoma *in situ* is histologically characterized by the proliferation of malignant epithelial cells that are bounded by the basement membrane of the breast ducts. This type of carcinoma is sometimes also characterized as pre-cancerous [29,30].

The carcinoma in mixed tumor exhibits a complex histological pattern showing components of epithelial and mesenchymal origin. The malignant epithelial cells exhibit infiltrative growth that can be identified by the loss of continuity of the basal/myoepithelial layer. The occurrence of non-invasive proliferation (*in situ*) can also be observed. The differences between *in situ* and invasive components are possible due to the presence of stromal invasion and microinvasion. The areas of invasion are characterized by the presence of clusters of infiltrative tumor epithelial cells in the regions of periductal stroma close to the components of the carcinoma. The microinvasion is identified in areas *in situ* with small regions where there is a projection of neoplastic cells that breaks the basement membrane [51]. In these carcinomas, it is possible to observe a greater organization of collagen fibres that make up the surrounding connective tissue in comparison to normal breast tissue. It is possible to observe the alignment of the collagen fibres of the connective tissue that surrounds the neoplastic growth.

The solid carcinoma is characterized by the proliferation of epithelial cells organized in a solid arrangement, with the formation of cords, sheets or agglomerates. The tumor cells are undifferentiated and exhibit small hyperchromatic nuclei with high mitotic index. The amount of stroma can vary from small to moderate and areas of necrosis are frequently observed [29,30]. As in the other carcinomas, it is also possible to observe greater organization of the collagen fibres at the surrounding connective tissue in comparison to normal breast tissue.

The collagen fibre extracted images highlight some of these features. The fibre angle distribution image (fourth row) for the normal tissue is clearly more colorful, since it is expected that the fibres are not well oriented, but dispersed in all directions along the connective tissue in the healthy mammary gland. Whereas for the images of the samples with cancer diagnoses some colors stand out in the image, indicating that most of the fibres are aligned in some specific directions. This indicates a higher organization of the collagen in the neoplastic tissues as was already discussed in the literature [4–12,17–21,23,25].

**Fig. 2.**
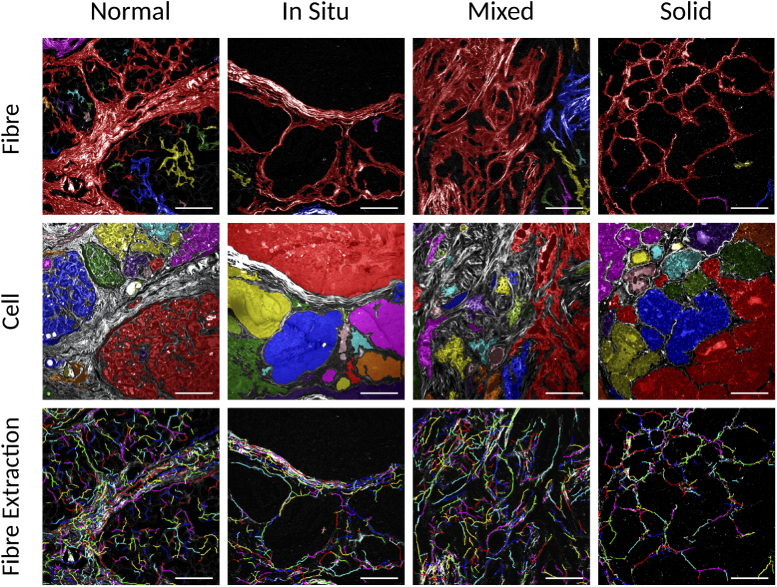
Extracted segments for the normal tissue and each cancer type indicated in the columns, as normal, *in situ*, mixed and solid, for the normal mammary gland, the carcinoma *in situ*, the carcinoma in mixed tumor and the solid carcinoma, respectively. The fibre segments in the first row, the cell segments in the second row and the third row shows the individual extracted fibres. The cell segments and the fibres are overlapped with the SHG intensity image in gray scale. The segments and the individual fibres are presented in a random color. The images are 0.471 mm × 0.471 mm with 512×512 pixels and the scale bar is 100 μm.

Based on the network extraction (Fig. 1 last row), it is possible to separate the regions of fibres and cells, and also to extract the single fibres from the images. Figure 2 shows examples of these extracted segments for the normal tissue and each cancer type as listed in the columns. The fibre segments are shown in the first row, the cell segments in the second row and the individual extracted fibres in the third row. The images are shown overlapped with the SHG intensity image in gray scale.

This segmentation allowed an unprecedented way to evaluate the properties specifically for the fibres and cell regions. The images show that the normal mammary tissue presents a larger number of fibre segments than the neoplastic tissue. This is an indication that the fibre segment is more connected in neoplastic samples. Important details evaluated by the pathologists for the diagnosis classification can be observed in these images. For the carcinoma in mixed tumor that presents characteristics areas of invasion, the invasion area can be clearly seen where there is a rupture of the fibre segment and thus the cell segment becomes larger. The cell segments merge into only one, where it would be expect to have a separation for an *in situ* classification. These extracted details allows a good separation between the histological cancer types.

### 3.1 Statistical analysis results

Here we present a discussion about a subset of metrics representing features of biopsy image that are considered by pathologists to be significant for the visual classification of neoplastic tissues. Some of the collagen parameters that were most discussed in the literature are also presented.

**Fig. 3.**
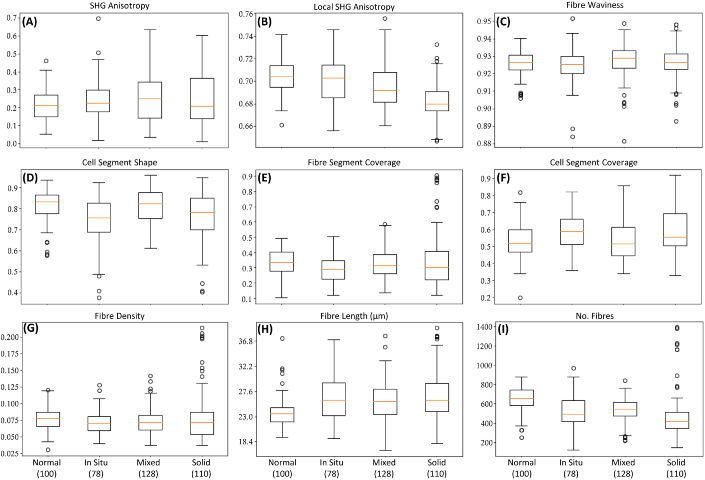
Boxplot graphics for the metrics indicated by the labels for the normal and tumor mammary tissues separated by the diagnosis: carcinoma *in situ*, carcinoma in mixed tumor and solid carcinoma. The numbers in parentheses are the number of acquired images for each histological type.

Figure 3 shows as boxplots the metrics obtained from the image analysis and Table 1 shows the p-value significance for the comparisons between diagnoses. Most of the metrics shows a good separation between the normal mammary gland and the other three tumor diagnoses, although no individual metric is significant enough to separate between all diagnoses. This highlights the importance of using our full set of 29 metrics to allow good discrimination of all cases studied as will be discussed in the next section.

**Table 1.** p value significance: p<0.05 are denoted as *, p<0.01 are denoted as ** and p<0.001 are denoted as ***.

Metrics	Normal	Normal	Normal	*in situ*	*in situ*	Mixed
	× *in situ*	× Mixed	× Solid	× Mixed	× Solid	× Solid
Total Anisotropy		**				
Local Anisotropy		***	***	*	***	***
Fibre Waviness				*		
Cell Segment Shape	***		***	***		***
Fibre Segment Coverage	**			**	*	
Cell Segment Coverage	***		***	*		**
Fibre Density	*					
Fibre Length	***	***	***			
No. Fibres	***	***	***			***

**SHG anisotropy** The total fibre anisotropy is calculated from the mean structure tensor of all pixels within a segment, n=f(⟨T(x,y)⟩). It measures the overall degree of fibre alignment and ranges from 0 (higher disorder, isotropic areas) to 1 (higher order, anisotropic areas). The Fiber Segment SHG Anisotropy therefore represents the average anisotropy calculated for all fibre segments in a SHG image. As shown in the boxplot, Fig. 3(A) and Table 1, there is a significant p value only between the diagnoses of normal mammary gland and the carcinoma in mixed tumor. One reason for not obtaining significant p values for the separation between the other diagnoses is due to the fact that, although in general the tumors show a larger fibre orientation, when analyzing the image as a whole the fibres still tends to organize around a lobe. Therefore, all directions of fibrous strong ordering will cancel each other out in the structure tensor, artificially reducing the total anisotropy of the region. Nonetheless, for the carcinoma in mixed tumor, there is a breakdown of the collagen fibres in some of the tumor borders allowing a different average anisotropy. These are features that compare well with the visual inspection for histological classification by the pathologists.

**Local SHG anisotropy** The local anisotropy is obtained for each pixel from the mean structure tensor of its 3×3 neighbouring pixel window, $n_{local}=\left\langle f\left(T(x,y)\right)\right\rangle$. It measures the degree of local alignment and also varies from 0 to 1 but, in comparison to the total anisotropy, it is not affected by the overall direction of pixels within each segment. The Fibre Segment SHG Local Anisotropy therefore represents the average local anisotropy calculated for all pixels in fibre segments of a SHG image. Figure 3(B) shows lower median values for the solid carcinoma and the carcinoma in mixed tumor in comparison with the median of the normal mammary gland diagnosis, showing a significant p-value for both comparisons. However for the normal mammary gland and carcinoma *in situ* the p value is not significant. For the other diagnoses the p values show significant differences for all cases, as can be seen in the Table 1.

**Fibre waviness** The fibre "waviness" can be measured as the ratio of fibre length to displacement, varying from 0 (infinitely wavy) to 1 (completely linear). Figure 3(C) shows that there is an increase in the waviness parameter for the neoplastic samples, which means that the fibres are more linear than those of normal tissue, that is in agreement with the literature [12,26]. But the median values for all the cancer types is very similar. The calculated p-values show significance only between the carcinoma *in situ* and the carcinoma in mixed tumor.

**Cell segment shape** This parameter is a simple measure of how circular or elongated a segment is. It is proportional to the ratio between the circumference of a circle with the same area as the segment and the segment perimeter, with values ranging between 0 (completely circular) and 1 (completely elongated). Therefore the Cell Segment Shape metric is the average "shape" of all extracted cellular segments in an image. The results are presented in Fig. 3(D) and in Table 1. The p values show significance for most of the comparisons.

It may be expected that normal tissue types will contain cellular regions that display more regular, circular appearance, whereas the value of the shape metric for this group suggests that they are more elongated. An explanation for this may be seen in Fig. 2; although visually the cellular regions of the normal type image (first column) are relatively circular, the colored cell segments identified by the analysis software are highly folded, due to the presence of the collagen structures within the regions that are detected by the SHG. This folding leads to a much greater value for the segment perimeter than would be expected by eye. In other tissue types, such as the *in situ* and solid carcinomas, the cellular regions display less collagen within the cell regions and so the cell segments are not folded. Therefore, a more sophisticated measurement of the cell segment shape could be employed in future to resolve these artifacts.

**Fibre segment coverage** The segment coverage represents the proportion of pixels containing the segment within the image. It is calculated by the ratio of fibre segment area to the total area of the segment bounding box. The results are presented in Fig. 3(E). The median values for all the carcinoma types decrease as compared to the normal tissue. This is in line with the pathologist observations, since for the tumor samples the cell proliferation compresses the extracellular tissue. The comparison between the types shows significant p-value between the normal tissue and the carcinoma *in situ* and also between the carcinoma *in situ* and all tumor types, see Table 1. It is interesting to note that the solid carcinoma has the lowest median value, as would be expected, since the tumor mass is mainly covered by cells [29,30].

**Cell segment coverage** The Cell Segment Coverage represents the proportion of the image covered by cell segments. It is calculated by the ratio of cell segment area to total area of segment bounding box. The results are presented in Fig. 3(F). The median values for all the carcinoma types are larger than the values for the normal mammary tissue and there is a significant p-value between most of the comparisons. As expected, this metric shows an opposite trend as the Fibre Segment Coverage (though this is not guaranteed by the segmentation process), with the median coverage value for the solid tumor being the largest among the histological types [29,30].

**Fibre density** We present an estimation of the fibre density by calculating the ratio of the SHG signal intensity in the fibre region of the image to the intensity in total image area. The results are presented in Fig. 3(G). The median values for the different diagnoses show a decrease in comparison to the normal mammary gland, but p values show significance only between the comparison of normal mammary gland and the carcinoma *in situ*, Table 1. These results are further indications of the decrease of the fibre area in comparison to the cell area in the carcinoma tissues [29,30].

**Fibre length** Another feature of interest is the average length of the extracted fibres, calculated from the Euclidean pixel distance along the fibre. The results are presented in Fig. 3(H). The median values for the carcinoma types are higher as compared to the normal mammary tissue, with a significant p-value between the normal tissue and all the carcinoma types (Table 1). It is worth noting that the length of fibres do not necessarily correlate with their waviness, though shorter fibres will likely be too constrained to display much difference between their length and displacement. These results are in agreement with the ones reported in the literature [31].

**Number of fibres** Average number of fibres extracted by the algorithm. The results are presented in Fig. 3(I). The median values for the carcinoma types are all lower as compared to normal mammary tissue. The solid carcinoma presents the lower values as expected from the characteristics of these tumors that show expansive cell growth in highly cellularized nests. The obtained p-values are significant for the comparisons of all cancer types to the normal mammary gland (Table 1). In addition, the comparison between the carcinoma in mixed tumor and the solid carcinoma also shows a significant p-value.

There appears to be a tendency in cancer type groups that contain a higher number of fibres to also possess a shorter average length, which at first may not seem obvious. However, when the fibre networks become more constricted and linear, the FiRE algorithm will have a greater tendency to consider adjoining fibres as connected during the extraction process. Therefore it seems appropriate that the carcinoma types with greatest progression report fewer, longer fibres alongside a lower fibrous segment coverage for the images.

### 3.2 LDA

**Fig. 4.**
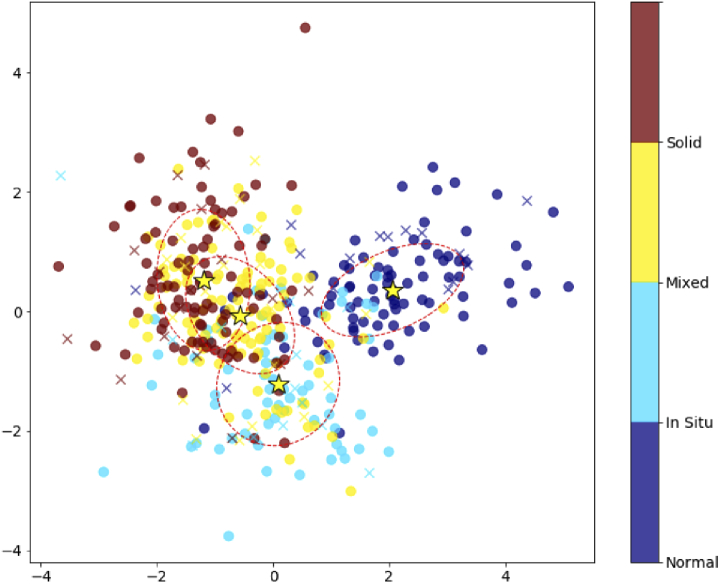
LDA plot considering all the metrics analyzed. The cases are indicated by the color bar. The full symbols are for the training set and the crosses are the LDA test set. The mean of each case group is indicated by the yellow star and the ellipses show one standard deviation of the training data.

In this section we present the LDA analysis considering the full set of 29 metrics. Figure 4 illustrates the LDA results for the two most discriminative components of each sample. It shows that the region of normal mammary tissues separates well from the tumor regions, as was already expected by the results presented in the boxplot graphics and the significant values of p. For the carcinoma in mixed tumor the ellipse shows a partial overlap with both carcinoma *in situ* and solid carcinoma ellipses. This is somewhat expected as for these tumors, the carcinomatous proliferation can exhibit *in situ* or infiltrative growth. Nevertheless, there is also a good separation for the carcinoma *in situ* and the solid carcinoma. This is an important finding, as the progression from carcinoma *in situ* to invasive carcinoma is one important step in mammary cancer evolution.

As expected from the overlap between carcinoma groups, our test dataset accuracy for separation between every diagnostic, 61±5.0%, is not optimal. However, when only evaluating the model prediction for images taken from normal tissue samples and the three types of carcinoma, we obtained an accuracy score of 91±2.8%. Therefore we consider the 29 image metrics as promising indicators of major stages in tumor progression, although further work is required in order to identify more subtle changes in the ECM between pre-cancerous and cancerous stages.

**Fig. 5.**
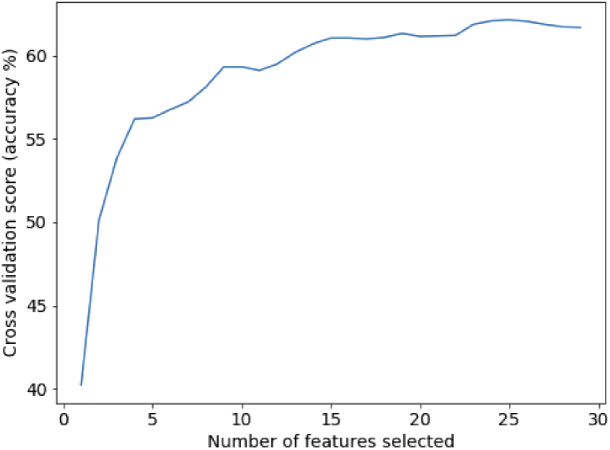
Cross-validations scores for an LDA model trained using stratified K-fold training set data, in relation to the number of metrics included in the model.

In order to investigate any redundancy in the 29 metrics, we also applied recursive feature elimination and cross-validation (RFECV) to keep only the relevant features in our LDA models. The performance of model CV scores, as we vary the number of features, is shown in Fig. 5. Although optimal CV scores can be reported for datasets containing less than 29 features, the overall difference in accuracy between models is negligible (±0.2%) compared to the variance between models trained from different folds. Therefore we retain the full set of metrics for any models reported on in this work.

## 4. Conclusion

In summary we used nonlinear microscopy techniques, second harmonic generation and fluorescence by absorption of two photons to analyze normal and tumor mammary tissues of female dogs. The image treatment based on the structural tensor and the extraction of networks, carried out by the PyFibre software, allowed to efficiently identify and separate the regions of fibres and cells in the images. By these analyses we were able to extract images showing the collagen single fibres, the fibre orientations and to quantify parameters specific for the fibre and cell regions in the images. The LDA results with the set of 29 metrics proved to be efficient in separating the samples of the normal mammary gland and the neoplastic tissues with a clear discrimination between the histological types. It should be noted that the same procedure can be used to diagnosis, as collected, fresh biopsies with no staining needed. The SHG imaging can be performed in thick fresh tissue cuts and the autofluorescence of the cells can also be used instead of the eosin fluorescence to identify the cell regions, that may provide a faster way to get a diagnosis.
